# Robot‐assisted Percutaneous Cannulated Screw Fixation of Femoral Neck Fractures: Preliminary Clinical Results

**DOI:** 10.1111/os.12430

**Published:** 2019-03-04

**Authors:** Sheng‐jun Duan, Hua‐shui Liu, Wen‐cheng Wu, Kun Yang, Zhen Zhang, Shi‐dong Liu

**Affiliations:** ^1^ Department of Traumatic Orthopaedics Affiliated Jinan Third Hospital of Jining Medical University Jinan China; ^2^ Intensive Care Unit People's Hospital of Feicheng Taian China

**Keywords:** Femoral neck fracture, Fracture fixation, internal, Robotics

## Abstract

**Objective:**

To assess the clinical efficacy of TiRobot‐assisted percutaneous cannulated screw fixation in the treatment of femoral neck fractures.

**Methods:**

From September 2015 to July 2017, 26 patients with unilateral femoral neck fractures were treated with TiRobot‐assisted percutaneous cannulated screw fixation. The femoral necks were fixed using three cannulated screws with robot assistance applying the following procedure: image acquisition, path planning, and needle and screw placement. The results of the treatment, including operation duration, frequency of fluoroscopy use, implant placement accuracy, intraoperative bleeding, total drilling, surgical complications, fracture healing time, fracture healing rate, and Harris scores at the last follow‐up, were recorded and compared with 23 similar patients who underwent conventional manual positioning surgery.

**Results:**

A total of 147 cannulated screws were placed in all patients. The TiRobot group had shorter operation duration (62.6 ± 8.7 min *vs* 72.4 ± 10.3 min) and fracture healing time (5.1 ± 2.4 months *vs* 5.9 ± 2.8 months) than the conventional group (*P* > 0.05). The robot group had significantly less use of fluoroscopy (26.5 ± 7.4 times *vs* 51.3 ± 9.4 times), intraoperative bleeding (8.2 ± 5.3 mL *vs* 36.4 ± 12.5 mL), and total drilling (9.4 ± 4.2 times *vs* 18.3 ± 9.1 times) than the conventional group (all *P* < 0.05). The screw parallelism was significantly improved (24.0 ± 0.6 points *vs* 21.5 ± 1.2 points) and the neck‐width coverage (72.0 ± 6.7 mm^2^ *vs* 53.8 ± 10.4 mm^2^) was significantly enlarged compared to the conventional group (*P <* 0.05). Only three guiding needles were used to penetrate the femoral head during manual insertion in the TiRobot group, which was significantly lower than that in the conventional group (3/78, 3.8% *vs* 9/69, 13.0%; *P <* 0.05). Other complications such as wound infection, vascular or nerve injury, screw loosening, and secondary screw displacement, did not occur in the two groups. There was no significant difference between the two groups in fracture healing rate (88.4% *vs* 82.6%) and Harris scores at the last follow up (88.2 ± 3.6 points *vs* 87.3 ± 4.7 points; *P* > 0.05).

**Conclusion:**

TiRobot‐assisted percutaneous cannulated screw fixation of femoral neck fractures is advantageous over conventional surgery with manual positioning due to easier manipulation, more accurate screw insertion, less invasion, and less radiation exposure, suggesting that it is a better method to stabilize femoral neck fractures.

## Introduction

Femoral neck fracture is the most common type of hip fracture and accounts for roughly half of all proximal femoral fractures. With the large increase in the number of geriatric patients worldwide, the incidence of femoral neck fractures is increasing yearly^1^, ^2^. Most fresh femoral neck fractures undergo anatomical reduction and internal fixation^3^. Timely surgery can prevent further fracture displacement, shorten the duration of convalescence, and reduce the incidence of complications^3^.

The most common treatment for femoral neck fractures is closed reduction and internal fixation with multiple cannulated screws. Minimally invasive three parallel cannulated screw fixation is an accepted method for the surgical treatment of medial femoral neck fractures^4^. Some studies have confirmed that exact screw placement enables a biomechanically stable fixation and reduces the risk of fracture nonunion^5^, ^6^. The conventional method of screw placement for femoral neck fractures is mainly performed by surgeons with experience in manual positioning under fluoroscopic monitoring. However, under X‐ray monitoring, ensuring the best position of each screw is difficult during surgery, and the accuracy varies due to different personal experience and inconsistency in operations. Moreover, repeated X‐ray exposure increases radioactive damage to patients and medical personnel.

In recent years, computer navigation or robot‐assisted minimally invasive internal fixation has been increasingly applied in orthopaedic surgery^7^, ^8^, ^9^. This method shows significantly better accuracy in positioning and less invasiveness, as well as shorter operation time and less radiation damage^10^, ^11^, ^12^, ^13^ compared with the conventional non‐navigated approach. Thus, it has been accepted by an increasing number of orthopaedic surgeons and promoted in clinical practice. Some studies have demonstrated that 3‐D computer‐assisted navigation significantly improves the accuracy of cannulated screw placement in the femoral neck^14^. The third‐generation orthopaedic surgery robot TiRobot, whose intellectual property rights are held in China, is the latest advanced orthopaedic robotic system. This robotic system uses a modular, small, and universal design. It has provided a breakthrough in 3‐D perspective navigation in surgery and extends indications for treatment to the spine and traumatic orthopaedic surgery, which helps surgeons complete the placement of cannulated screws efficiently and safely, with the positioning accuracy reaching 0.6–0.8 mm.

Most hospitals still use free hand empirical screw placement for femoral neck fixation under fluoroscopic monitoring. Following the introduction of the TiRobot system in our hospital, we performed robot‐assisted cannulated screw fixation of femoral neck fractures and obtained some preliminary clinical results. The objectives of this prospective study were: (i) to investigate the clinical efficacy of TiRobot‐assisted femoral neck surgery; (ii) to discuss the advantages of this surgery; and (iii) to summarize the surgical precautions and limitations of robot‐assisted femoral neck surgery.

## Methods

### *Inclusion and Exclusion Criteria*

The inclusion criterion was any adult patient with unilateral closed femoral neck fractures. Exclusion criteria were patients who: (i) were >65 years of age with Garden fracture classification type III or type IV; (ii) could not undergo closed reduction because of multiple injuries or severe trauma; and (iii) could not tolerate the operation because of comorbidities.

### *Patients’ Information*

This prospective study reviewed a case series from September 2015 to July 2017. The study protocol was approved by our Institutional Review Board. Informed consent was obtained from all participants included in the study. A total of 49 patients were admitted in this study and underwent percutaneous cannulated screw fixation of femoral neck at the authors’ institution.

A total of 26 patients ranging in age from 26 to 82 years (11:15 of male‐to‐female ratio and 61.7 ± 5.2 years of mean age) were treated with TiRobot‐assisted percutaneous cannulated screw fixation. The causes of injury were: 14 cases of slip and fall injury, 7 cases of traffic injury, 3 cases of sports injury, and 2 cases of high‐level fall injury. According to the Garden classification, there were 3 type I, 7 type II, 10 type III, and 6 type IV fractures. The mean time from injury to operation was 6.3 days (range, 1–18 days).

Twenty‐three patients who had undergone conventional surgery with manual positioning during the same time period were selected as the control group. They were 9 men and 14 women ranging in age from 24 to 84 years (mean age, 62.1 ± 4.1 years). The causes of injury were: 13 cases of slip and fall injury, 6 cases of traffic injury, 3 cases of sports injury, and 1 case of high‐level fall. According to the Garden classification, there were 2 cases of type I, 7 cases of type II, 9 cases of type III, and 5 cases of type IV. The time from injury to operation was 1 to 16 days.

Patient characteristics are presented in Table 1. There was no statistically significant difference in general characteristics between the two groups (all *P* > 0.05).

**Table 1:** General characteristics of the two groups

Patient characteristics	TiRobot group (26 cases)	Conventional group (23 cases)	*P‐*value
Age (years, mean± SD)	61.7 ± 5.2	62.1 ± 4.1	0.727
Gender (cases)			0.821
Female	15	14	
Male	11	9	
Injury side (cases)			0.907
Left	14	12	
Right	12	11	
Injury cause (cases)			0.967
A	14	13	
B	7	6	
C	3	3	
D	2	1	
Garden classification (cases)			0.984
I	3	2	
II	7	7	
III	10	9	
IV	6	5	
Time from injury to operation (days, mean ± SD)	6.3 ± 2.3	6.5 ± 2.4	0.753

A is a slip and fall injury; B is a traffic injury; C is a sports injury; D is high‐level fall injury. SD, standard deviation.

### *Surgical Equipment and Instruments*

**Figure 1:**
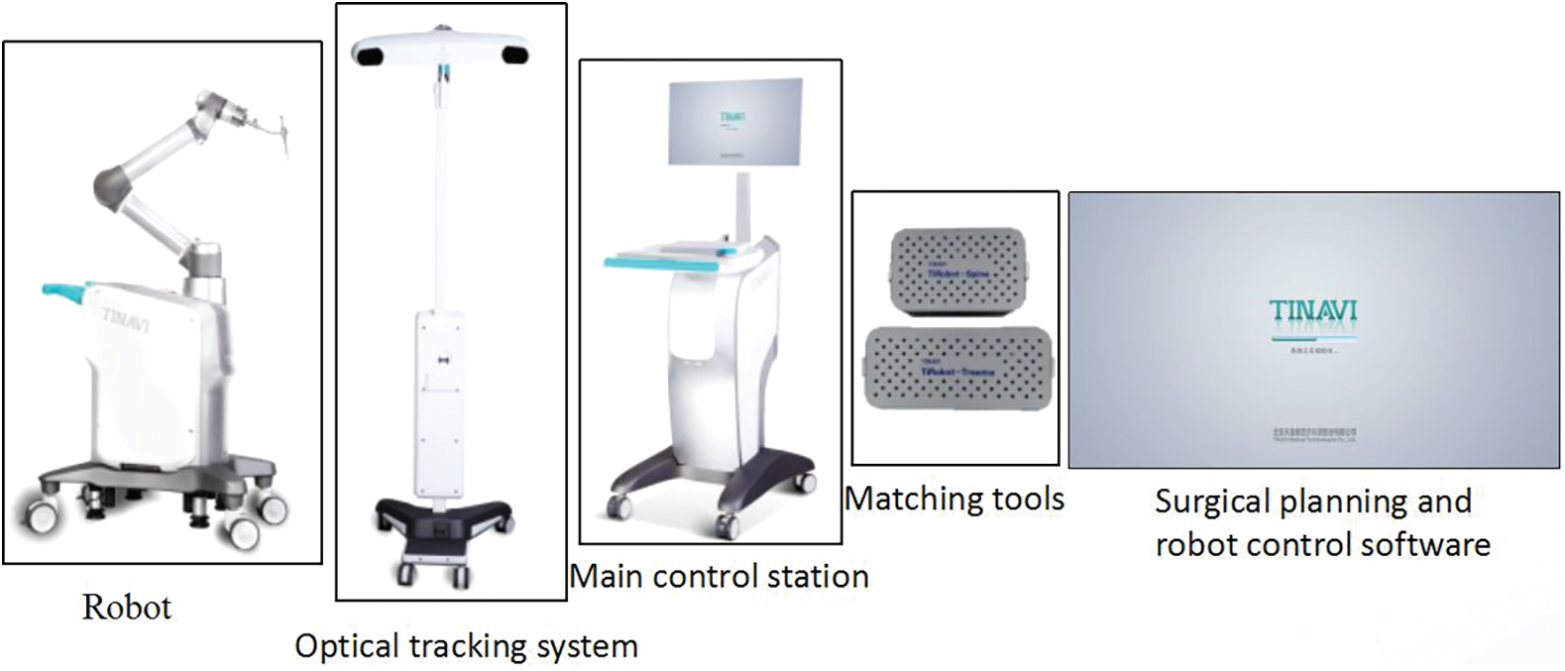
The composition of the TiRobot system.

The TiRobot system (TINAVI Medical Technologies, China), the third generation of the TIANJI orthopaedic robot, consists of a robot, spatial calibration components, surgical planning, robot control software, an optical tracking system, a main control station, and matching tools (Fig. 1). We used the C‐arm X‐ray system (Siemens, Germany), an orthopaedic traction bed, a guiding needle with a diameter of 3 mm and length of 400 mm, and AO 7.3‐mm‐diameter cannulated screws (Tianjin Zhengtian Medical Instruments, China).

### *Surgical Procedure (TiRobot Group)*

#### *Starting System*

We connected the robot, the main control station, and the C‐arm fluoroscope system through the data line. We connected the power source, started the control station, logged in to the surgical planning software, and entered the patient information.

#### *Install the Scaler*

We selected the corresponding positioning scaler according to the injury side, assembled with the robot and fastened tightly. We completed the scale calibration according to the software prompts.

#### *Patient Preparation*

Under combined spinal and epidural anesthesia, the patients were placed in supine position on the orthopaedic traction bed, with the injured limb fixed with continuous traction. Anteroposterior (AP) and lateral C‐arm X‐ray fluoroscopy was performed to examine the effects of reduction. If the reduction was satisfactory, routine disinfection was performed.

#### *Equipment Layout*

The main station and the C‐arm X‐ray monitor were placed outside the healthy side and facing the operator. The robot positioning system was placed on the outside of the injured limb. The surgical bed was adjusted to the appropriate height and the C‐arm X‐ray machine was placed between the lower limbs. The scaler should be faced to the patient’s head. The long axis of the positioning system was required to be parallel with the longitudinal axis of the affected femur. The frontal center hole of the position scaler should be located in the center of the femoral neck, and the lateral center hole is required to be the same height as the femoral neck.

#### *Image Acquisition*

For 3‐D image acquisition, the C‐arm was positioned isocentrically to the femoral neck, in both AP and lateral projections. All 10 locating points of the scaler were required to be clear and distinguishable in the AP and lateral image. Then, the images were transferred to the system.

#### *Path Planning*

The AP and lateral images of X‐ray fluoroscopy were imported to the software of the platform. The surgeon marked the locations of the three screws in the software interface according to the patient’s anatomical features and fracture status. The system automatically generated three virtual screws in the interface. When the three screws presented as an “inverted triangle” layout, the implant positions were considered satisfactory. The software automatically calculated the screw length as a reference.

#### *Surgical Operation*

**Figure 2:**
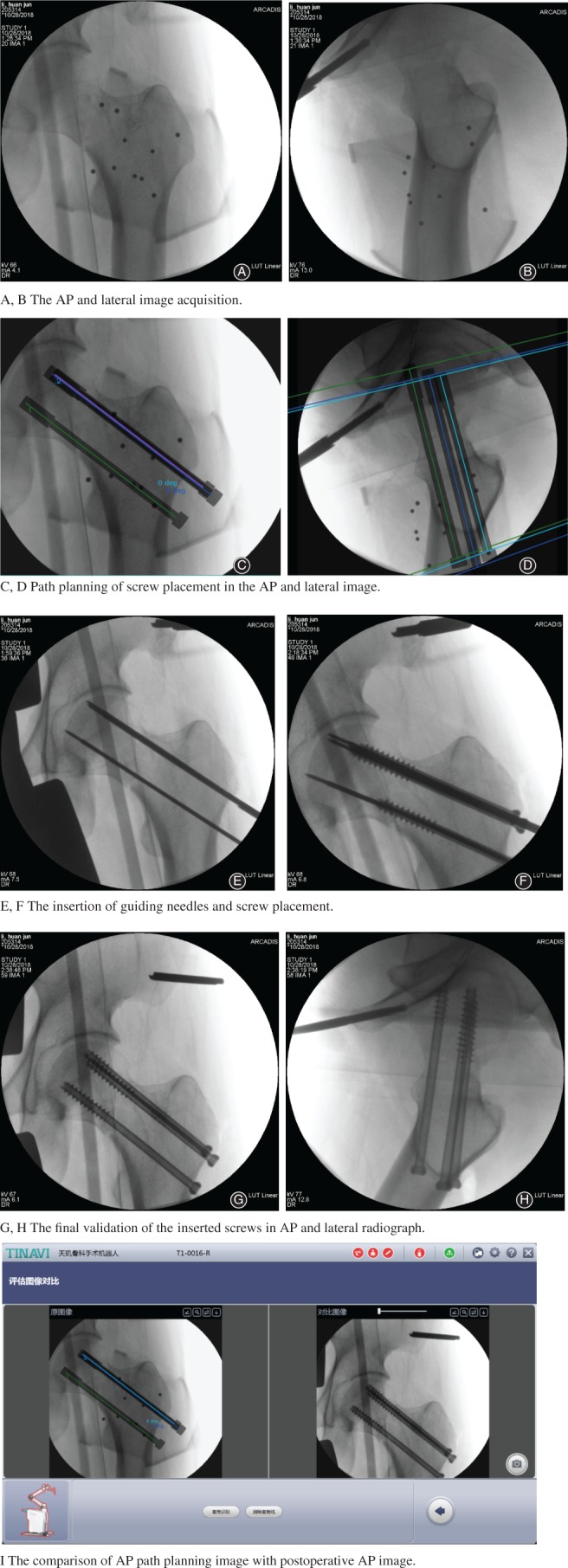
A 45‐year‐old man with a left femoral neck fracture. The surgical procedure of TiRobot‐assisted percutaneous cannulated screw fixation. (A, B) The anteroposterior (AP) and lateral image acquisition. (C, D) Path planning of screw placement in the AP and lateral image. (E, F) The insertion of guiding needles and screw placement. (G, H) The final validation of the inserted screws in AP and lateral radiograph. (I) Comparison of AP path planning image with postoperative AP image.

The surgeon selected the first guiding needle in the software and clicked the “move” button, and the system automatically controlled the robotic arm to move to the planned entry point. The drill sleeve was installed and placed into the positioning slot. The drill sleeve was then moved close to the skin. A 2‐cm stab incision was made, the subcutaneous tissue and fascia were bluntly separated, and the drill sleeve was inserted until the tip was pushed tightly on the bone surface where the screw would be placed. The position and direction of the sleeve was checked by AP and lateral X‐ray fluoroscopy. If the sleeve position was found to be biased, we used the tuning button for alteration until its position was correct. A 3‐mm‐diameter guiding needle was then inserted into the drill sleeve. The calculated screw length in the software can be a reference in the process of insertion, and a special depth gauge was used to measure the depth of the needle at the same time. The position of the needle was once again confirmed through X‐ray when it was inserted. The sleeve was extracted and the excess needle outside the skin was cut off. The same method was used to insert the second and the third guiding needles. Afterwards, we removed the robot, expanded the appropriate canal with a cannulated drill bit, and three cannulated screws with a diameter of 7.3 mm and a length of 80–100 mm were screwed in through each guiding needle in the following order: below screw, front screw, and rear screw. The screws were examined by fluoroscopy again. Finally, the guiding needles were removed and the subcutaneous tissues and the skin were closed. Figure 2 shows a patient with a left femoral neck fracture to illustrate the surgical procedure.

### *Surgical Procedure (Conventional)*

Three cannulated screws were inserted into femoral necks guided by conventional fluoroscopic imaging. The surgeon used a C‐arm fluoroscope in conventional 2‐D mode. The guiding needles were repeatedly adjusted for insertion location and angle, and were gradually advanced using a cannulated single drill guide under repeated optimal image intensification in two planes until the subchondral bone of the femoral head was reached. They were placed cranially in the femoral neck with their distribution presented as an “inverted triangle” layout. After manually measuring the length, a cannulated drill bit was used to drill the appropriate canal. Three 7.3‐mm‐diameter cannulated screws were then inserted over the guiding needles. Then, the guiding needles were removed and the subcutaneous tissues and the skin were closed.

### *Postoperative Treatment and Follow‐up*

The postoperative regimens were similar between groups. Prophylactic anti‐infection treatment was used for 24 h after the surgery. Meanwhile, the AP pelvic radiographs and the lateral affected hip radiographs were repeated. Based on standard postoperative AP and lateral images, the screw implant placement accuracy required screw parallelism, and the neck‐width coverage triangular area was evaluated according to the model of Hamelinck *et al*.^15^. At 24 h after the surgery, the patients were assisted with gentle passive hip flexion activities 2–3 times a day and were instructed to carry out lower‐limb strength training. The patients were allowed to perform proactive bending of the hip and the knees in bed 2 weeks after the surgery. After 4 weeks, the patients could perform non‐weight‐bearing movements with the help of a walker. The X‐ray exam was repeated monthly after surgery. If after 3 months the fracture line was obviously blurred, the patients were allowed to perform partial weight‐bearing movements with the help of a walker. After 6 months, patients could attempt full weight‐bearing walking when X‐ray imaging showed that the fracture was healed. The fracture healing rate and adverse events were recorded, including femoral penetration, wound infection, fixation loosening, fragment re‐displacement, and femoral head necrosis. The Harris hip score system was used to assess the hip function outcome at the last follow‐up.^16^

### *Statistical Analysis*

Quantitative data are expressed as mean ± standard deviation (SD) and were compared using the *t*‐test. Categoric variables were compared by Pearson χ^2^‐test. All analyses were performed with SPSS 17.0 (SPSS, Chicago, IL). *P*‐values less than 0.05 were considered significant.

## Results

### *General Results*

A total of 147 cannulated screws were placed in all patients. The operation duration (from starting the system to closing the skin) of the TiRobot group was 55–115 min, with an average of 77.3 min, and there was no significant difference compared with the conventional group, which had an average operation duration of 79.0 min (range, 60–110 min, *P* > 0.05). The fluoroscopy frequency was 22–40 times in the TiRobot group, with an average of 28.6 times. Intraoperative bleeding in the TiRobot group ranged from 5 to 15 mL, with an average of 9.5 mL. Seventy‐five screws were successful placed on the first try in the TiRobot group. The total drilling times of the TiRobot group was 3–7 times, with an average of 4.3 times. The corresponding results of fluoroscopy frequency, intraoperative bleeding, and total drilling times in the conventional group were 46.7 times, 41.3 mL, and 18.1 times on average, respectively, which was significantly different from the TiRobot group (*P* < 0.01). A comparison of results is shown in Table 2.

**Table 2:** Comparison of results in the two groups (mean ± standard deviation)

Results	TiRobot group (26 cases)	Conventional group (23 cases)	*P*
Operation duration (min)	77.3 ± 9.3	79.0 ± 9.8	0.547
Fluoroscopy frequency (number)	28.6 ± 9.6	46.7 ± 8.5	<0.001
Intraoperative bleeding (mL)	9.5 ± 6.8	41.3 ± 12.4	<0.001
Total drilling times (number)	4.3 ± 1.8	18.1 ± 7.2	<0.001
Screw parallelism (points)	24.0 ± 0.6	21.5 ± 1.2	<0.001
Triangular area (mm^2^)	72.0 ± 6.7	53.8 ± 10.4	<0.001
Femoral penetration (number)	3	9	0.041
Fracture healing (number)	23	19	0.559
Fracture healing time (months)	4.6 ± 1.9	5.3 ± 2.1	0.223
Harris score (points)	88.3 ± 4.4	87.6 ± 3.9	0.559

### *Clinical Complications and Outcomes*

Three guiding needles were used to penetrate the femoral head during manual insertion in the TiRobot group (3.8%), and nine were used to penetrate the femoral head in the conventional group (13.0%). There was a significant difference in the penetration rate in the two groups. All wounds healed as intended. All the patients were available at a mean follow‐up of 13.6 months (range, 5–26 months). Thirty‐three fractures in the TiRobot group and 19 in the control group healed in a good position. There were no complications such as wound infection, vascular or nerve injury, screw loosening, or secondary screw displacement. The average fracture healing time was 4.6 months (range, 4–8 months) in the TiRobot group and 5.3 months (range, 4.5–9 months) in the conventional group, and the difference was not significant (*P* > 0.05). The fracture healing rate in the TiRobot group (88.4%) was not significantly difference from that of the conventional group (82.6%, *P* > 0.05). There was also no significant difference between the two groups in Harris scores at the last follow‐up (88.3 ± 4.4 points *vs* 87.6 ± 3.9 points, *P* > 0.05).

### *Accuracy of Screw Placement*

For the screw implant placement accuracy in the TiRobot group, as shown in Table 2, the screw parallelism was significantly improved (24.0 ± 0.6 points *vs* 21.5 ± 1.2 points) and neck‐width coverage was significantly enlarged (72.0 ± 6.7 mm^2^ *vs* 53.8 ± 10.4 mm^2^) compared to the conventional group (*P* < 0.05).

## Discussion

With the development and updating of medical imaging and computer technologies, computer‐assisted orthopaedic surgery (CAOS) has been widely used in joint surgery, spine surgery, and traumatic orthopaedics^7^, ^8^, ^9^, ^17^. The stereotactic technique based on X‐ray or 3‐D CT images can assist doctors to perform more accurate surgical planning, improve accuracy, avoid the errors of manual operation, and reduce radiation exposure^10^, ^11^, ^12^, ^13^, ^18^. With the continuous development and functional improvement of various orthopaedic robot systems, robot‐assisted minimally invasive orthopaedic surgery has been accepted by an increasing number of orthopaedic doctors and promoted in clinical practice.

### *Safety and Efficacy of TiRobot‐assisted Femoral Neck Surgery*

In this study, the TiRobot system almost successfully assisted percutaneous cannulated screw fixation of femoral neck fractures on the first try with intuitive surgical path planning. In fact, multiple cannulated screw fixation is a safe surgical procedure for stabilizing femoral neck fractures. There were no complications such as wound infection, vascular or nerve injury, screw loosening, or secondary screw displacement in our study. The mean operation duration of the TiRobot group was 77.3 ± 9.3 min, which is not significantly different from that of the conventional group. The total operation duration included the non‐invasive period of robot path planning and the invasive period of the actual operation. In fact, the duration of the surgery from the moment of inserting the first guiding needle until the skin was closed was only 10–20 min, which was significantly less than in the conventional group. Because we performed this operation relatively quickly and with limited experience, most of the time was spent on equipment placement and debugging, image acquisition, and other non‐invasive procedures. When the surgeon has more experience with the planning steps, the total operation duration might be reduced.

The number of drill attempts should be kept to a minimum as they can weaken the cortical and cancellous bone and might lead to subtrochanteric fractures^19^. In the present study, the total number of holes drilled in the TiRobot group was significantly lower than that in the conventional group, and precise positioning was achieved on the first attempt during the operation, avoiding repeated drilling, in contrast to the traditional surgery. The reduced number of total holes drilled resulted in less trauma due to drilling attempts and reduced the intraoperative bleeding.

Extensive use of fluoroscopy may endanger the patient and the operating room staff. In terms of radiation exposure, the mean number of intraoperative fluoroscopies in the TiRobot group was 28.6, significantly less than that in the conventional group, and was also far below the number of reports in some studies^1^ with conventional manual positioning, heralding a significant reduction in the radiation damage to medical staff and patients.

The goals for screw positioning were to obtain the maximal spread of three parallel screws in the femoral neck through the formation of an inverted triangle with the apex at the femoral calcar, and to avoid femoral head penetration. Exact screw placement with a greater distance between the screws enables a biomechanically stable fixation. The X‐ray images of all the patients showed that all three cannulated screws achieve the “inverted triangle” effect. Our study found that screw parallelism was significantly improved and neck‐width coverage significantly increased with the use of TiRobot navigation. This has been shown to improve the stability of fracture fixation^4^, ^20^. Twenty‐three patients’ fractures had healed at last follow up. The fracture healing rate was higher than in the conventional group, but the difference was not statistically significant. There was no difference in fracture nonunion and other adverse events between groups. The type of fracture, the defect of the screw fixation itself, and early inappropriate functional exercise may be the causes of the nonunion. Of course, assessment of long‐term complications such as ischemic necrosis of the femoral head requires longer periods of follow up. The present research will be supplemented and improved in further studies with increased number of patients.

### *Advantages of TiRobot‐assisted Femoral Neck Surgery*

There is a relatively wide range of indications for TiRobot‐assisted percutaneous cannulated screw fixation of femoral neck fractures. In fact, the present study revealed that TiRobot‐assisted surgery did not increase the fracture healing rate and functional scores. However, the main advantages may be summarized as follows. The first advantage is accurate positioning. The robot provides precise spatial positioning and stable path navigation for the placement of the screw. Through the movement of the robotic arm, screws were guided and placed accurately, safely, and stably in the anatomical sites. A second advantage is programmed surgical procedures. The procedure only required collection of 2‐D X‐ray images, and the software provided prompts to complete the path planning and drilling positioning. The three guiding needles can be precisely inserted at the planned position as long as the procedures are closely followed. A third advantage is having a correction function. If the actual path of the guiding needle is found to deviate from the planning path, the angle of the mechanical arm can be adjusted using the fine adjustment function of the software, so as to effectively ensure the safety of the operation. A fourth advantage is reduced radiation damage. Compared with manual screw placement, robotic navigation significantly reduced the intraoperative cumulative radiation dosage.

### *Surgical Precautions*

Surgical precautions may be summarized as follows. First, unskilled manipulation can lead to prolonged non‐invasive time. The positioning is accomplished by space mapping, operation planning, and surgical path localizing. The surgeon should be familiar with the equipment, have repeated practice, and accumulate experience to increase the smoothness of the operation and to shorten the overall operation time. Second, the path planning of screw placement still relies on the experience of surgeons, and there may be subjective errors. Third, a drill sleeve that is too long with high lateral stress may lead to the deviation of the tip of the guiding needle. Fourth, the cost of equipment ($2 million) is relatively high and needs special personnel training.

### *Study Limitation*

This study is only a single center’s preliminary results and there is a lack of multi‐center controlled studies. Further research is needed with prospective multi‐center randomized controlled studies with a large number of patients and long follow‐up.

## Conclusions

Minimally invasive multiple cannulated screw fixation is an accepted method for stabilizing femoral neck fractures. The TiRobot provides precise spatial positioning and stable path navigation for screw placement of femoral neck fractures, which overcomes the shortcomings of the conventional methods, such as unstable manual operation, visual deviation and fatigue, and more radiation damage. It can achieve satisfactory clinical results with higher accuracy, less invasion, and less radiation exposure, suggesting it is a better method for minimally invasive treatment of femoral neck fractures.
